# A seamless Phase I/II platform design with a time-to-event efficacy endpoint for potential COVID-19 therapies

**DOI:** 10.1177/09622802241288348

**Published:** 2024-10-14

**Authors:** Thomas Jaki, Helen Barnett, Andrew Titman, Pavel Mozgunov

**Affiliations:** 1 Faculty for Informatics and Data Science, University Regensburg, Germany; 2 MRC Biostatistics Unit, 2152 University of Cambridge, UK; 3 School of Mathematical Sciences, Lancaster University, UK

**Keywords:** Adaptive platform trial, dose-escalation, COVID-19, randomized, seamless, time-to-improvement

## Abstract

In the search for effective treatments for COVID-19, the initial emphasis has been on re-purposed treatments. To maximize the chances of finding successful treatments, novel treatments that have been developed for this disease in particular, are needed. In this article, we describe and evaluate the statistical design of the AGILE platform, an adaptive randomized seamless Phase I/II trial platform that seeks to quickly establish a safe range of doses and investigates treatments for potential efficacy. The bespoke Bayesian design (i) utilizes randomization during dose-finding, (ii) shares control arm information across the platform, and (iii) uses a time-to-event endpoint with a formal testing structure and error control for evaluation of potential efficacy. Both single-agent and combination treatments are considered. We find that the design can identify potential treatments that are safe and efficacious reliably with small to moderate sample sizes.

## 1. Introduction

The emergence of COVID-19 and the ensuing pandemic has led to a widespread, frantic, search for treatments. Despite large uncertainty about the underlying pathogen and the natural history of the disease, trials must start rapidly to identify treatments to save lives, but also so that effective treatments can be used in the response to the outbreak. A consequence of this is that trials in COVID-19 in the first few weeks and months of the outbreak have focused on re-purposed treatments.^1–3^

While recently, some success with using re-purposed treatments has been achieved,^4–6^ it is crucial that development of treatments specifically developed for COVID-19 is also undertaken in order to maximize the chances of finding therapies to successfully treat patients. The crucial difference of trials investigating novel therapies (in contrast to re-purposed treatments) is that the range of safe and likely effective doses is unknown. Therefore, an efficient dose-finding design identifying safe and active doses to be studied in larger trials is essential. While there exist a number of dose-finding designs for early phase dose-finding trials evaluating toxicity and efficacy simultaneously, for example, Wages and Tait^7^ and Mozgunov and Jaki^8^ and references therein, many of them consider a binary efficacy endpoint with few recent extension to other endpoints.^9–11^ Time-to-event endpoints with censoring at 28 days have previously been used as a clinically meaningful measure in a number of COVID-19 trials^1,2,4^ and the argument has been made that they should be considered in all COVID-19 trials.^12^

While the majority of Phase I dose-finding trials, particularly in oncology, are non-randomized, it is agreed that in later phases, the gold standard for evaluating novel treatments are well-conducted blinded randomized controlled clinical trials. At the same time, in light of the uncertainty about the symptoms caused by COVID-19 – especially at the beginning of the pandemic – it is essential to conduct randomized dose-finding trials to ensure that the risk of adverse events is correctly attributed to the drug under study rather than to the disease itself. Moreover, it has been argued that adaptive designs^13,14^ are particularly suitable during a pandemic, also in the light of the uncertainty about a novel disease.^15^ Therefore, a randomized adaptive dose-finding design evaluating both toxicity and time-to-event efficacy would allow to answer the research questions of interest in novel therapies for treating COVID-19.

It is also recognized that there are a number of novel therapies that have the potential to be efficient in fighting COVID-19. Therefore, it is crucial to have a structure in place that allows rapid enrolment of novel therapies to ensure rapid decision-making, and, importantly, would allow for efficient use of information between the studies, that is, utilizing the data from the control treatment across different compounds. This can be achieved via a platform trial.^16^

**Figure 1.**
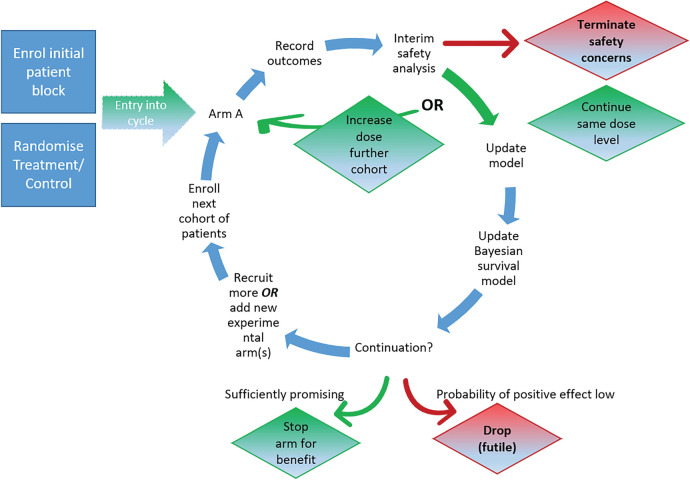
Illustration of the AGILE platform design.

In this paper, we describe and evaluate the bespoke design developed and implemented for the AGILE platform,^17^ an adaptive randomized seamless Phase I/II dose-finding trial platform that seeks to quickly establish a safe range of doses and investigates treatments for potential efficacy using a Bayesian sequential trial design (see a visualisation of the design for one compound in Figure 1). The proposed design is unique as it

(i) utilizes randomization during dose-finding to allow COVID-19 induced symptoms to be distinguished from drug side-effects,
(ii) shares control arm information across the platform in order to maximize efficiency, and
(iii) uses a time-to-event endpoint with a formal testing structure and error control for evaluation of potential efficacy,

making the design particularly suitable for the pandemic setting. We also extend the design for trials studying dual-agent combinations of treatments.

The rest of the article is organized as follows. Section 2 describes the platform for single treatments while its performance is evaluated in simulations in Section 3. The design for dual-agent combinations is proposed in Section 4 and subsequently evaluated in Section 5. We conclude with a discussion (Section 6).

## 2. Single-agent design

### 2.1. Setting

Consider a randomized controlled dose-escalation clinical trial in which m increasing doses $d_{1}<d_{2}<\cdots <d_{m}$ of a single experimental treatment are studied. Let $d_{0}=0$ be a dose of zero of the treatment, which is subsequently referred to as the *control arm* (or, simply control). The inclusion of the control arm is motivated by the emerging nature of the symptoms associated with COVID-19 when planning the study and the desire to avoid labelling potential treatments as unsafe due to misclassifying non-treatment-related symptoms. Note that information on the control arm can come from either patients randomized to control for this particular candidate treatment within the platform, or from the control arm of other candidates within the platform provided that they are concurrent. A binary outcome of a random variable Y , y=0 is observed if no dose-limiting event (DLE) is observed within $t_{\mathrm{safe}}>0$ days after randomization, and y=1 , otherwise. Let $p_{j}$ be the probability for a patient to experience a DLE if given dose $d_{j}$ . It is assumed that the risk of DLE is a non-decreasing function of dose, $p_{0}\leq p_{1}\leq \cdots \leq p_{m}$ and prior information for the DLE probability of the control arm, $p_{0}$ , is available.

As it is expected that the control arm is associated with a non-negative (unknown) risk of DLE (or symptoms of the disease that cannot be distinguished from DLEs), the primary goal of the dose-escalation is formulated in terms of the additional risk of a dose-limiting event (ADLE) defined in terms of the expected difference in DLE risk between the doses of the agent and the control. Specifically, we therefore seek to identify the dose that corresponds to an additional risk of γ=0.20 which equates to finding the dose $d_{j^{⋆}}$ such that

$$\begin{array}{r} j^{⋆}=\arg\;\min_{j=0,\ldots ,m}|\left(p_{j}-p_{0}\right)-\gamma | \end{array}$$

### 2.2. Bayesian dose-escalation model

The following randomized Bayesian dose-escalation design that builds on the proposal by Mozgunov et al.^18^ is used. Assume that the DLE probability has the functional form

(1) $$\begin{array}{r} \varphi ({\tilde{d}}_{j},\theta_{1},\theta_{2})=\frac{\exp\;(\theta_{1}+\theta_{2}{\tilde{d}}_{j})}{1+\exp\;(\theta_{1}+\theta_{2}{\tilde{d}}_{j})} \end{array}$$

where $\theta_{1}$ and $\theta_{2}$ are unknown parameters, and ${\tilde{d}}_{j}$ is a standardized dose level (also referred to as a skeleton) corresponding to dose j , which is constructed given the information about the prior DLE toxicities (details are given below). We require that $\theta_{2}>0$ and enforce this constraint through the construction of the prior distribution (see Section 3.3.1). This model choice was found to result in good statistical properties in terms of the target dose identification in a randomized dose-finding trial.^18^ We require that the standardized dose level corresponding to control is equal to ${\tilde{d}}_{0}=0$ . This will guarantee that a sequential update of the slope parameter $\theta_{2}$ will not contribute to the DLE probability estimation on the control arm yet all data are used for its estimation.^18^

Denote the prior distribution of the vector $\theta =(\theta_{1},\theta_{2})$ by $f_{0}(.)$ . To construct the standardized levels, ${\tilde{d}}_{j}$ , we represent them in terms of prior estimates of the DLE probabilities ${\hat{p}}_{j}^{\left(0\right)}$ associated with doses $d_{j}$ j=0,…,m

(2) $$\begin{array}{r} {\tilde{d}}_{j}=\frac{\mathrm{logit}({\hat{p}}_{j}^{\left(0\right)})-{\hat{\theta }}_{1}^{\left(0\right)}}{{\hat{\theta }}_{2}^{\left(0\right)}} \end{array}$$

where ${\hat{\theta }}_{1}^{\left(0\right)}$ and ${\hat{\theta }}_{2}^{\left(0\right)}$ are prior point estimates of the model parameters, and logit(x)=log(x/(1−x)) is the logit transformation of x . To satisfy ${\tilde{d}}_{0}=0$ , the prior needs to be chosen such that $\mathrm{logit}({\hat{p}}_{0}^{\left(0\right)})={\hat{\theta }}_{1}^{\left(0\right)}$ .

Assume that n patients, potentially including concurrent control patients from other evaluations within the platform, have already been assigned to doses $\tilde{d}(1),\ldots ,\tilde{d}(n)$ and binary responses $Y_{n}=[y_{1},\ldots ,y_{n}{]}^{T}$ were observed, respectively. The model updates the posterior distribution of θ using Bayes’ theorem

(3) $$\begin{array}{rl} f_{n}(\theta ) & =\frac{f_{n-1}(\theta )\phi (\tilde{d}(n),y_{n},\theta )}{\int_{R^{h}}f_{n-1}(u)\phi (\tilde{d}(n),y_{n},u)du}\\ & =\frac{f_{0}(\theta )\prod_{i=1}^{n}\phi (\tilde{d}(i),y_{i},\theta )}{\int_{R^{h}}f_{0}(u)\prod_{i=1}^{n}\phi (\tilde{d}(i),y_{i},u)du} \end{array}$$

where $\phi (d(n),y_{n},\theta )=\varphi (d(n),\theta {)}^{y_{n}}(1-\varphi (d(n),\theta ){)}^{1-y_{n}}.$ This posterior distribution is then used to make the escalation/de-escalation decision.^19^ Specifically, the first set of safe doses is defined as the doses $d_{j}$ for which

(4) $$\begin{array}{r} P\left(p_{j}-p_{0}\geq \gamma +2\delta \right)<c_{\mathrm{overdose}} \end{array}$$

where γ is the target ADLE risk, δ is the width of the interval of DLE risk which we consider acceptable, $c_{\mathrm{overdose}}$ is the threshold controlling overdosing, and the probability is found with respect to the updated posterior distribution. Amongst the safe doses, the dose which maximizes

(5) $$\begin{array}{r} P\left(p_{j}-p_{0}\in [\gamma -\delta ,\gamma +\delta ]\right) \end{array}$$

is selected as the target dose.

### 2.3. Efficacy design

#### 2.3.1. Bayesian efficacy model

In this study, we assess the potential efficacy of the treatment for a particular dose instead of modelling efficacy across all doses. Although other approaches are possible, our approach allows us to make conclusions about a given dose alone without sharing information from other arms and enables control of the type I error for the assessment of a given dose. A Cox proportional hazards model is assumed where the hazard of recovery at time t is given by $h(t;z)=h_{0}(t;z)\psi^{z}$ , where z=1 corresponds to a treatment and z=0 to control. We use a two-point prior for ψ (detailed below) for computational efficiency and a parametric model is assumed in order to maximize power in the light of small sample sizes and in the absence of a clear understanding of potential reasons for deviation from the proportionality assumptions. We study the impact of violating this assumption in a sensitivity analysis presented in the supplemental materials. Initially, the cohort of patients who have graduated from the dose-escalation stage are followed up for a total of $t_{\mathrm{eff}}$ days. Based on their outcomes, a decision is made to either stop for futility, stop for efficacy or recruit a further cohort of patients. To improve power, controls recruited from other candidate treatments or other doses of the same treatment are also used within the evaluation, but this is restricted to using only the most recent, concurrent, $n_{c}$ such controls to mitigate the risk of bias due to population drift.

A Bayesian criterion is adopted for the stopping rule at each stage. In line with Bayesian thinking, we set the stopping rules to be the same for each stage k . Specifically denoting all data up to stage k on dose j by $D_{k}^{\left(j\right)}$ and for a given desirable treatment effect, $\psi^{*}$ ,

- evaluation is stopped for efficacy if $\pi_{E|k}^{\left(j\right)}:=P(\psi =\psi^{*}|D_{k}^{\left(j\right)})>u$ ,
- evaluation is stopped for futility if $\pi_{E|k}^{\left(j\right)}<l$ , or
- an additional cohort of patients is recruited, otherwise.

In order to ensure a decision is made at the final stage k=K , efficacy for dose $d_{j}$ is established if $\pi_{E|K}^{j}>u$ and is considered futile otherwise. This is in line with traditional group-sequential designs where the lower and upper boundaries are made equal at the final analysis.

A point prior of the form $P(\psi =1)=1-\pi_{E}^{\left(j\right)},\;P(\psi =\psi^{*})=\pi_{E}^{\left(j\right)}$ is assumed for ψ . Here $\pi_{E}^{\left(j\right)}$ represents the degree of optimism or scepticism towards the likely efficacy of dose $d_{j}$ .

An advantage of the point prior is that obtaining the posterior probability $\pi_{E|k}^{\left(j\right)}$ is computationally straightforward allowing comprehensive evaluations of the design via simulations. The posterior probability under this model is

(6) $$\begin{array}{r} \pi_{E|k}^{\left(j\right)}=\frac{\pi_{E}^{\left(j\right)}L(\psi^{*}|D_{k}^{\left(j\right)})}{\pi_{E}^{\left(j\right)}L(\psi^{*}|D_{k}^{\left(j\right)})+(1-\pi_{E}^{\left(j\right)})L(1|D_{k}^{\left(j\right)})} \end{array}$$

where $L(u|D_{k}^{\left(j\right)})$ is the Cox partial likelihood with respect to the data for dose j up to the k th period evaluated at a hazard ratio of u . While the Cox partial likelihood is often not considered compatible with a Bayesian analysis since it does not use the full information of the data, Bayesian justifications for its use are available.^20,21^

#### 2.3.2. Setting the boundaries

To set the boundaries, (l,u) , a large number of trajectories of $L(1|D_{k}^{\left(j\right)})$ and $L(\psi^{*}|D_{k}^{\left(j\right)})$ can be simulated under both the null and alternative hypothesis, where in all cases the simulation continues until the maximum period K . An assumption regarding the proportion of patients recovered by time $t_{\mathrm{eff}}$ under the null is needed, in addition to the hazard ratio between treatments. In the absence of censoring due to drop-out, the results will be otherwise invariant to the precise survival distribution assumed since the Cox partial likelihood only uses the order of events.

The effect of varying (l,u) can then be explored by converting the pairs of likelihoods into a posterior probability and imposing the boundary-stopping rules. For any given set of boundaries, the type I error, power, expected number of patients under the null, expected number of patients under the alternative and probabilities of stopping for futility or efficacy at each stage, can be approximated. The boundaries can then be set to optimize some criterion, for instance sum of expected sample sizes under the null and alternative, subject to some constraints, for instance controlling type I error, keeping power above some level or limiting the chance of early stopping under the alternative.

The inclusion of historic controls will increase both the power and type I error of any procedure, for example, Schmidli et al.^22^ To ensure type I error is controlled for the evaluation of the given dose, the boundaries are set assuming the maximum, $n_{c}$ , previous controls are available, with the consequence that the type I error will be lower for the first treatment evaluated in the platform. This will also mean the power will be lower for the first few evaluated treatments. However, given the dose-finding design, it is anticipated that the first evaluations will be of less importance, as for safety reasons evaluations tend to start at sub-optimal low doses.

### 2.4. Overall design

The overall design of the platform allows for multiple different compounds to be evaluated and, by sharing concurrent control group data, efficiency is gained. For any compound in the platform, patients are allocated in cohorts of size $c=c_{1}+c_{2}$ , where $c_{1}$ is the number of patients in the cohort assigned to an active dose and $c_{2}$ is the number of patients in cohort assigned to the control arm, ${\tilde{d}}_{0}$ , throughout its evaluation. Below is an outline of the overall procedure for one compound made up of both safety and efficacy evaluation:

Safety evaluation

1. The first cohort of $c_{1}+c_{2}$ patients is assigned to the first dose and to the control arm, respectively.
2. After $t_{\mathrm{safe}}$ days, short-term DLE outcomes are collected and the posterior distribution of the parameters is updated using equation (4).
3. The set of safe doses is found using equation (4).
  - If no doses are safe, the trial is stopped for safety;
  - if only the current dose is safe, the next cohort of $c_{1}+c_{2}$ patients is assigned to the current dose and to the control arm, respectively;
  - otherwise, the next cohort of $c_{1}+c_{2}$ patients is assigned to the adjacent, safe dose level for which the probability (5) is maximized and control arm, respectively, that is, no skipping of dose levels is allowed.
4. Once efficacy information is available for two cohorts on a safe dose, that dose is graduated to the efficacy evaluation.

Efficacy evaluation

1. If a dose $d_{j}$ is deemed safe, the efficacy outcome is observed up to day, $t_{\mathrm{eff}}$ .
2. The posterior probability, $\pi_{E|k}^{\left(j\right)}$ , following equation (6) is then computed where k corresponds to the number of times this dose has been evaluated for efficacy.
  - If $\pi_{E|k}^{\left(j\right)}<l$ evaluation of dose $d_{j}$ is stopped for futility;
  - if $\pi_{E|k}^{\left(j\right)}>u$ evaluation is stopped for efficacy and the corresponding candidate and dose $d_{j}$ recommended for further testing;
  - otherwise if k<K , an additional cohort of $c_{1}+c_{2}$ patients is recruited on the current dose and control arm, respectively.

The evaluation of a dose continues until the maximum number of patients N on a dose has been reached unless it is stopped for efficacy, futility or safety before. Once all doses are stopped, the evaluation of this candidate stops. Note that this structure implies that different doses of one compound that are deemed safe can independently be evaluated against control in this design. The overall design for one compound is depicted in Figure 1.

## 3. Evaluations of proposed design

### 3.1. Setting

We will now evaluate, for one compound, safety and efficacy across the study together in a simulation study and evaluate the impact of shared control data that are gradually accumulated over the course of the trial, thereby assessing the added benefit of the platform structure. To our knowledge there are no alternative approaches that (i) utilize randomization during dose-finding, (ii) assess efficacy using a time-to-event endpoint within a formal testing framework and (iii) employ a platform structure and hence no comparator is presented here. Our sensitivity analysis presented in the supplemental materials, however, provides a comparison with a design that uses a similar design with a binary outcome for efficacy.

We consider the setting where there are three active doses ( m=3 ) of a single agent and the control arm. As before, the DLE endpoint is binary and corresponds to either experiencing or not experiencing a DLE by time $t_{\mathrm{safe}}=7$ and the efficacy endpoint is time-to-improvement defined as a 1-category improvement on the 10-point World Health Organization scale^23^ over 28 days, $t_{\mathrm{eff}}=28$ . To generate the efficacy outcome a Weibull distribution is used. For the control group, the rate and shape parameters were 0.085 and 0.797, respectively, resulting in a median recovery time of 14 days and a recovery rate of 70% within 28 days. The rate parameter for the candidate dose has been adapted to match the scenarios described below. The binary safety and time-to-event efficacy responses are assumed to be highly correlated and are generated via a procedure described by Mozgunov et al.^24^ using a correlation coefficient of ρ=0.8 .

The maximum total intake per dose level is 72 patients assigned to each dose level and control, which equates to a maximum total sample size of 216. In line with the real study, cohort sizes of $c_{1}=4$ (assigned to active doses) and $c_{2}=2$ (assigned to the control) are used. Note that imbalanced randomization between active doses and control is used here, as additional control information from different doses of that compound and from concurrently available control data from other compounds is also used in the efficacy assessment. We also report sensitivity analysis to assess the impact of various cohort sizes.

The objective of the trial is to find all safe efficacious doses to be graduated into a larger Phase II or Phase III clinical trial. The target ADLE risk is γ=0.20 , and the dose is considered safe if the ADLE risk is ≤0.30 ( δ=0.05 ) and is efficacious if it corresponds to a hazard ratio of at least 1.75. The stopping boundaries for efficacy have been found such that the type I error in each pairwise comparison (i.e. for each dose compared to control) is controlled at 10% one-sided, while the power for each individual dose is 80%. The resulting boundary values are l=0.224 and u=0.839 (see Section 3.3.2 for more details).

### 3.2. Scenarios

As the trial aims to study novel compounds which have yet to be explored with respect to their mechanism of action in COVID-19 patients, it is crucial that the design has good operating characteristics under a variety of dose-DLE and dose-efficacy scenarios. Therefore, we consider five dose-efficacy scenarios ranging from no doses corresponding to a change in time-to-improvement within 28 days to all doses resulting in a clinically significant reduction; and five dose-DLE scenarios ranging from all doses being safe to all doses being very unsafe. We then consider all combinations of these scenarios, resulting in 25 scenarios explored in total. The five dose-DLE and dose-efficacy scenarios for each ( $d_{0}$ , $d_{1}$ , $d_{2}$ , $d_{3}$ ) are presented in Table 1.

**Table 1.** Safety and efficacy scenarios for ( $d_{0}$ , $d_{1}$ , $d_{2}$ , and $d_{3}$ ).

	Safety (probability of DLE, $p_{j}$ )	Efficacy (hazard ratio)
Scenario 0	(0.10, 0.12, 0.13, 0.15)	(1.00, 1.00, 1.00, 1.00)
Scenario 1	(0.10, 0.12, 0.15, 0.30)	(1.00, 1.00, 1.75, 1.75)
Scenario 2	(0.10, 0.15, 0.30, 0.45)	(1.00, 1.50, 1.75, 1.75)
Scenario 3	(0.10, 0.30, 0.45, 0.60)	(1.00, 1.50, 1.75, 2.00)
Scenario 4	(0.10, 0.45, 0.60, 0.60)	(1.00, 1.75, 2.00, 2.00)

DLE: dose-limiting event.

We will refer to the scenario with dose–DLE relationship x and dose–efficacy relationship y as ‘Scenario x to y ’. Each dose under the combination of DLE and efficacy scenario is classified as incorrect, undesirable, acceptable or desirable. If a treatment is unsafe or has a hazard ratio of 1 then it is classed as incorrect. If it is safe, then a hazard ratio of 1.25 is undesirable, 1.5 is acceptable and at least 1.75 is desirable.

For all 25 scenarios, a sensitivity analysis is conducted on varying values of $c_{1}$ and $c_{2}$ in order to assess the effect of both altering the allocation ratio between control and active doses and the total cohort size, c . We also study the implications of not sharing controls across doses. A total of six settings are considered, ( $c_{1}=2$ & $c_{2}=1$ and $c_{1}=2$ & $c_{2}=2$ for only the settings where controls are shared, and $c_{1}=4$ & $c_{2}=2$ and $c_{1}=3$ & $c_{2}=3$ for both settings where controls are shared and where they are not). The maximum number of cohorts per dose varies with cohort size in order to maintain the constant maximum total intake per dose level of 72 across that dose level and control.

Software in the form of R code used to produce the presented results is available on GitHub (https://github.com/dose-finding/covid19-agile).

### 3.3. Choice of design parameters

#### 3.3.1. Safety model

The proposed design requires the prior and design parameters for both safety and efficacy parts to be pre-specified in advance of the conduct of the trial. The procedure of how these parameters were chosen is given below.

The prior parameters for the safety model were obtained via a calibration procedure^25^ over a number of safety scenarios (not taking into account efficacy). We use safety Scenarios 1 to 3 in Table 1 that correspond to the target dose being $d_{3}$ , $d_{2}$ , and $d_{1}$ , respectively, thus covering various locations of the target dose on the dosage grid.

The following prior distribution for the vector of safety model parameters θ was assumed:

$$\begin{array}{r} \left(\theta_{1},\log\;(\theta_{2}))∼N(\mu ,\Sigma \right) \end{array}$$

where $\mu =(\mu_{1},\mu_{2}{)}^{T}$ is the vector of means and

$$\begin{array}{r} \Sigma =\left[\begin{array}{cc} \sigma_{1} & \sigma_{12}\\ \sigma_{12} & \sigma_{2} \end{array}\right] \end{array}$$

Given the link between the prior toxicity on the control and the intercept parameter $\theta_{1}$ as implied by equation (2), $\mu_{1}=\mathrm{logit}({\hat{p}}_{0}^{\left(0\right)})$ where the prior DLE probability at the control, ${\hat{p}}_{0}^{\left(0\right)}$ . Following discussions with the clinical team, the DLE risk on control was set to ${\hat{p}}_{0}^{\left(0\right)}=0.10$ . To reduce the computational complexity of the calibration, the covariance between the model parameters was assumed to be $\sigma_{12}=0.$ The rest of the parameters were chosen by conducting simulations using various combinations of values of the parameters on the grid, $\mu_{2}=\{-0.5,-0.25,0,0.25,0.5\},\sigma_{1}=\{1.2,1.3,1.4,1.5,1.6\},\sigma_{2}=\{0.15,0.25,0.35,0.45,0.55\}.$

Furthermore, to define the standardized doses ${\tilde{d}}_{j}$ in equation (2), the prior toxicity probability at each dose should be assumed. As there is no reliable information on the DLE rates in the COVID-19 population, the skeleton was also calibrated. The grid of values for the prior toxicity risks is chosen in terms of the difference in the probability of DLE between the neighbouring doses. Specifically, ${\hat{p}}_{j}^{\left(0\right)}={\hat{p}}_{0}^{\left(0\right)}+\nu \times j,j=1,2,3$ where ν is the difference in the toxicity probabilities between doses, which are then used to find the skeleton using equation (2). The grid of values of ν was included as one of the parameters for the calibration ν={0.075,0.100,0.125,0.150} . Below, we fix $c_{\mathrm{overdose}}=0.25$ that was previously found to result in good safety properties of the design for the two-parameter logistic model.^26^

The calibration was performed as follows. For each combination of parameters of $\nu ,\mu_{2},\sigma_{1},\sigma_{2}$ on the specified grid, 500 simulations were run under each of the three considered scenarios monitoring the proportion of target dose selections. Then, the selected values of the parameters are those that maximized the geometric mean (taken across scenarios for the same combination of values of the parameters) of the proportion of the target dose selection. This resulted in using $\nu =0.125,\mu_{2}=-0.25,\sigma_{1}=1.40,\sigma_{2}=0.35$ for the further design evaluation.

#### 3.3.2. Efficacy model

The efficacy stopping boundaries for a particular setting were taken as the pair (l,u) that maximizes

(7) $$\begin{array}{r} \text{Criterion}=\text{Power}-\lambda \left\{\frac{E(N_{0})+E(N_{1})}{2*\max N}\right\} \end{array}$$

subject to the constraint that the type I error is ≤ 10%. The maximum total sample size, maxN , is 216 here, and $E(N_{0})$ and $E(N_{1})$ are the expected sample sizes (across both active dose and control arms) under the null and alternative hypothesis, respectively. The tuning parameter, λ , controls the contribution to the criterion such that λ>1 implies more emphasis on expected sample sizes and λ<1 more weight on power.

A value of λ=1.35 was chosen to allow a power of 80% to be achieved when ψ=1.75 for the main settings where $n_{c}=30$ and $c_{1}:c_{2}=4:2$ . For comparability, the same λ was used in the sensitivity analysis where the size of cohorts and/or the use of past controls was varied. In these other settings, power is lower than 80%, ranging from 63% to 79%. The boundaries for the scenarios are shown in Table 2. We can see from these results that a 2:1 allocation within a compound is more powerful than an equal allocation. This is due to the inclusion of additional concurrent control patients (up to 30) from within the platform.

**Table 2.** Boundaries for settings in the sensitivity analysis of cohort sizes. The settings have the same maximum sample sizes and common criteria to trade-off power and average sample size.

Cohort						
$c_{1}$	$c_{2}$	$n_{c}$	l	u	Power	Criterion
4	2	30	0.224	0.839	0.800	0.630
3	3	30	0.268	0.841	0.747	0.573
2	1	30	0.192	0.858	0.794	0.634
2	2	30	0.227	0.858	0.744	0.566
4	2	0	0.317	0.815	0.634	0.438
3	3	0	0.271	0.821	0.691	0.484

Throughout, the point prior is taken to be $\pi_{E}^{\left(j\right)}=1/2$ for all doses j . Note that if the boundaries are chosen based on (7), the operating characteristics of the efficacy design are not affected by the choice of point prior. Specifically, since (6) can be re-expressed in terms of the posterior log-odds as

$$\begin{array}{r} \log\;\left(\frac{\pi_{E|k}^{\left(j\right)}}{1-\pi_{E|k}^{\left(j\right)}}\right)=\log\;\left(\frac{\pi_{E}^{\left(j\right)}}{1-\pi_{E}^{\left(j\right)}}\right)+\log\;\left(\frac{L(\psi^{*}|D_{k}^{\left(j\right)})}{L(1|D_{k}^{\left(j\right)})}\right) \end{array}$$

changing the value of $\pi_{E}^{\left(j\right)}$ merely has the effect of translating the posterior log-odds by a constant. Hence the boundaries $\left(\tilde{l},\tilde{u}\right)$ under the alternative prior will satisfy the relationship $\left(\text{logit}(\tilde{l}),\text{logit}(\tilde{u}))=(\text{logit}(l)+\xi ,\text{logit}(u)+\xi \right)$ , where ξ is the log-odds ratio between the new and old prior odds of efficacy.

### 3.4. Results

**Figure 2.**
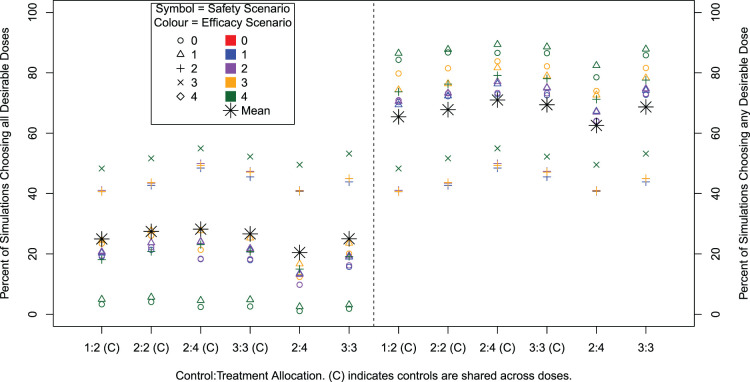
Percentage of simulations that recommend all desirable doses (left) and the percentage of simulations that recommend any desirable dose (right) for different cohort sizes and compositions and with and without sharing control group data. Note that only 13 out of 25 efficacy/safety scenarios contain a desirable dose.

**Figure 3.**
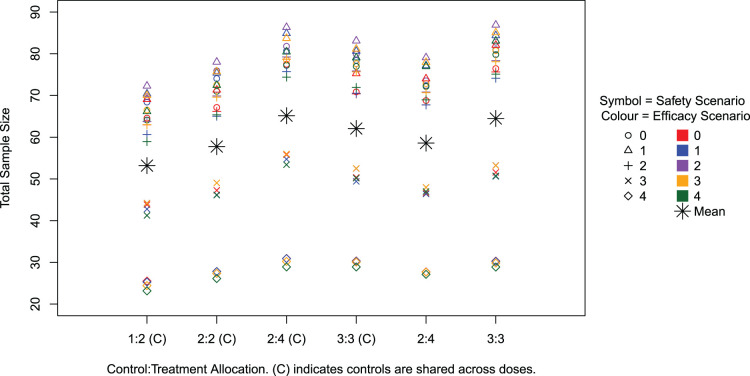
Average total sample size across simulations for all scenarios.

Detailed results for the setting with a cohort size of 4+2 and inclusion of up to 30 control patients are presented in Table 3. A comparison of results for the varying cohort sizes is illustrated in Figures 2 and 3. Across settings, the overall type I error rate, that is the percentage of simulations in Scenario 0–0 where any dose is recommended, ranges from 11% to 14%. Note that, for a given dose level, the type I error is controlled at 10% as desired and we observe only a small increase when we take all possible doses into account.

**Table 3.** Percentage of 10,000 simulations where each dose is recommended for ( $d_{1}$ , $d_{2}$ , $d_{3}$ ) for $c_{1}=4$ and $c_{2}=2$ , with controls shared across doses.

	Efficacy scenario					
		0	1	2	3	4
**Safety**	**0**	(0.4, 2.3, 8.9)	(0.4, **24.0**, **67.2**)	(*2.9*, **24.2**, **67.5**)	(*2.9*, **24.6**, **80.6**)	(**5.1**, **29.7**, **80.9**)
	**1**	(1.1, 5.9, 8.0)	(1.2, **47.4**, **52.9**)	(*8.6*, **47.6**, **53.6**)	(*9.1*, **47.3**, **62.1**)	(**14.0**, **56.3**, **61.6**)
**Scenario**	**2**	(5.5, 7.9, 2.4)	(5.7, **48.4**, 9.4)	(*31.7*, **49.9**, 9.8)	(*31.4*, **49.3**, 10.9)	(**45.0**, **57.2**, 10.9)
	**3**	(8.4, 2.1, 0.1)	(8.2, 7.3, 0.2)	(*40.4*, 7.8, 0.2)	(*40.4*, 7.9, 0.2)	(**55.0**, 8.3, 0.2)
	**4**	(5.0, 0.1, 0.0)	(4.9, 0.4, 0.0)	(17.6, 0.3, 0.0)	(17.6, 0.3, 0.0)	(23.1, 0.4, 0.0)

Desirable doses are highlighted in **bold** and acceptable doses are highlighted in *italics*. Note that these may sum to more than 100% for each scenario as more than one dose can be recommended simultaneously.

Figure 2 shows the percentage of simulations where all desirable doses are recommended (left) and where any desirable doses are recommended (right). For the baseline setting of $c_{1}=4$ and $c_{2}=2$ , the mean percentage of simulations recommending all desirable doses is 28.2%, whilst 71.0% of simulations recommended any desirable dose. As expected, safety/efficacy scenarios where only one dose is desirable have higher percentages of simulations recommending all desirable doses, whilst lower percentages of simulations recommending any desirable dose. The highest power for recommending any desirable dose is observed in the safety/efficacy scenarios where all active doses are safe and efficacious, reaching 89% for Scenarios 1 to 4.

The sensitivity analysis across the varying cohort settings shows there is a very small difference in performance. The ordering of performance across safety/efficacy scenarios is identical with only a small numerical difference. However, we can see that not sharing controls decreases the performance. Power for recommending any desirable dose increases for increasing cohort size c , on average increasing 6% for allocation 1:2 ( c=3 and c=6 ) and 2% for allocation ratio 1:1 ( c=4 and c=6 ). Again unsurprisingly, a lower power is observed when controls are not shared, and within this a higher power when the allocation ratio between control and active dose within cohort is equal. The highest power is achieved for cohort sizes $c_{1}=4$ and $c_{2}=2$ with controls being shared.

Figure 3 illustrates the average total sample size across the scenarios and settings. On average about 65 patients are required in the setting used in the trial with the total sample size exceeding 150 in only 1% of simulations across scenarios. The scenarios with the smallest sample sizes are those where all doses are unsafe and the trial is therefore stopped early for safety. In such cases, it takes 30 patients across 6 weeks on average to reach the conclusion of stopping early for safety. The scenarios with larger sample sizes are those where all doses are safe and most are acceptable or only just desirable (i.e. not the case where the hazard ratio is 2.00), as in these cases more doses are taken to the efficacy part and more patients are required to detect the smaller difference in hazard ratios.

It can be seen across settings that the larger the total cohort size, the larger the total sample size. This also corresponds to the higher power settings. When controls are shared, altering the control : treatment allocation from 1:1 to 1:2 decreases the total average sample size, whereas when controls are not shared this increases. This also links to the relationship with the power in the corresponding settings; a higher power is achieved for equal allocations when controls are not shared. The sharing of controls means more patients can be allocated to the novel treatment and hence more can be learnt about it. In all cases, the average total sample size is below 90.

Table 3 gives more detail into which doses are recommended across simulations. For example, in efficacy Scenario 2, where the lowest dose is acceptable and the higher two are desirable in terms of efficacy. In safety Scenario 0, where all doses are safe, the highest dose is chosen most often. In safety Scenario 2 where only the highest dose is unsafe, the middle dose is chosen most often, although less often than the highest desirable dose in Scenario 0. In safety Scenario 4 where all doses are unsafe, the lowest dose is chosen only 17.6% of the time. It is clear that desirable doses are recommended most often, with incorrect and undesirable doses rarely recommended. This gives insight that the procedure is successful in identifying desirable doses of a single agent.

Our additional sensitivity analyses investigating the violation of proportional hazards and providing a comparison against a binary efficacy outcome, presented in the supplemental materials, show that the design is fairly robust to violations of the proportional hazards assumption. Major violations of this assumption yield increased power at the expense of higher type I error. At the same time, these analyses show that the proposed time-to-event approach is superior to using a binary efficacy endpoint as expected.

## 4. Extension to combination treatments

### 4.1. Setting

Consider now a randomized controlled dose-escalation dual-agent clinical trial studying the combinations of J doses $d_{1}<d_{2},\ldots ,d_{J}$ of the first compound (referred to as agent A ) and of L doses $s_{1}<s_{2}<\cdots <s_{L}$ of the second compound (referred to as agent B ). As before, let $d_{0}=s_{0}$ be a zero dose of each compound, respectively, correspondingly to the control treatment, and denote the combination of dose $d_{j}$ of A and dose $s_{l}$ of B by $\left(d_{j},s_{l}\right)$ . Within each agent (assuming the second agent is fixed), it is known prior to the trial that the risk of a DLE monotonically increases with the dose. The objective of the trial is then to study the safety of the combinations and to establish the maximum tolerated combination, the combination corresponding to the target ADLE over the control arm of 20%. Denoting the probability of DLE at combination with doses $d_{j}$ and $s_{l}$ by $p(d_{j},s_{l})$ , the probability of each agent given individually by $p(d_{j})$ and $p(s_{l})$ , and the probability of DLE at the control by $p(d_{0},s_{0})=p_{0}$ , the aim is to find the combination $\left(d_{j^{⋆}},s_{l^{⋆}}\right)$ minimising

$$\begin{array}{r} |\left(p(d_{j},s_{l})-p_{0}\right)-\gamma | \end{array}$$

The fundamental difference to the single-agent setting introduced in Section 2.1 is that one cannot order all of the combinations of the compounds with respect to the monotonically increasing risk of DLE despite the monotonicity assumption being satisfied within each compound. For example, comparing combination $\left(d_{1},s_{2}\right)$ and $\left(d_{2},s_{1}\right)$ , the dose of one agent is increased and another is decreased, and it is unknown prior to the trial which of these effects prevails in the overall DLE risk associated with the combinations. Consequently, the model-based design for monotherapies in Section 2.2 cannot be used as an alternative dose-finding. Below, the extension of the single-agent model-based design is suggested.

### 4.2. Dual-agent Bayesian dose-escalation model

For the considered randomized dual-agent combination setting, under the assumption of independence of the compounds, the probability of a DLE associated with combination $\left(d_{j},s_{l}\right)$ can be written as

(8) $$\begin{array}{r} p_{0}(d_{j},s_{l})=1-(1-p(d_{j}))(1-p(s_{l})) \end{array}$$

To allow for the interaction of the compounds in terms of the probability of a DLE, we use the proposed model by Neuenschwander et al.^27^

$$\begin{array}{r} \mathrm{odds}(p(d_{j},s_{l}))=\mathrm{odds}(p_{0}(d_{j},s_{l}))\times \exp\;\left(\eta \times {\tilde{d}}_{j}\times {\tilde{s}}_{l}\right) \end{array}$$

where odds(p)=p/(1−p) is the odds transformation of the probability p , and η is the interaction coefficient, positive values of which correspond to synergistic DLE risk, zero corresponds to additive effect without interaction, and negative values correspond to the antagonistic risk of DLE, and ${\tilde{d}}_{j}$ , ${\tilde{s}}_{l}$ are standardized dose levels corresponding to the same DLE probability as the doses $d_{j},s_{l}$ .

Note that p(⋅) in equation (8) is the probability of a DLE associated with one compound given as monotherapy as in Section 2.2. Therefore, we adopt the two-parameter logistic model given in equation (1) for each agent separately. Specifically, let

(9) $$\begin{array}{r} p(d_{j})=\varphi ({\tilde{d}}_{j},\theta_{1},\theta_{21}) \end{array}$$

and

(10) $$\begin{array}{r} p(s_{l})=\varphi ({\tilde{s}}_{l},\theta_{1},\theta_{22}) \end{array}$$

where $\theta =(\theta_{1},\log\;(\theta_{21}),\log\;(\theta_{22}),\eta )$ are the unknown parameters with a normal prior distribution, θ∼N(μ,Σ) where $\mu =(\mu_{1},\mu_{21},\mu_{22},\mu_{\eta }{)}^{T}$ is the vector of means and

$$\begin{array}{r} \Sigma =\left[\begin{array}{cccc} \sigma_{1} & \sigma_{1,21} & \sigma_{1,22} & 0\\ \sigma_{1,21} & \sigma_{21} & 0 & 0\\ \sigma_{1,22} & 0 & \sigma_{22} & 0\\ 0 & 0 & 0 & \sigma_{\eta } \end{array}\right] \end{array}$$

As before, we require the standardized dose level corresponding to the control treatment to be equal to ${\tilde{d}}_{0}={\tilde{s}}_{0}=0$ , so that the intercept parameter of the two-parameter model (8) relates to the probability of DLE on the control only. Therefore, both single-agent models employ the same intercept parameter $\theta_{1}$ as it corresponds to the probability of a DLE at the same control treatment. Consequently, for small to moderate values of probability of DLE, the intercept parameter in the single-agent model, $\theta_{1}$ approximately equals the logit inverse-logit transformation of the half of the probability of DLE on the control treatment subject $\mathrm{logit}(p(d_{0},s_{0})/2)=\theta_{1}$ . This is used to construct the standardized dose levels ${\tilde{d}}_{j}$ , ${\tilde{s}}_{l}$ using the prior means of the parameters θ and the prior probabilities of a DLE at each combination similarly to the construction in equation (2).

Parameters of the vector θ are the unknown quantities that define the combination–DLE relationship. As in the single-agent design in Section 2.2, the posterior distribution of these is sequentially updated using the data collected during the trial using Bayes’ theorem. Specifically, denote the joint prior distributions of vector θ by $f_{0}(.)$ . Assume that n patients have received the combinations (d1),s(1))…,(d(n),s(n)) and binary responses $Y_{n}=[y_{1},\ldots ,y_{n}{]}^{T}$ were observed, respectively. The models update the posterior distribution of θ as

(11) $$\begin{array}{rl} f_{n}(\theta ) & =\frac{f_{n-1}(\theta )\phi ((d(n),s(n)),y_{n},\theta )}{\int_{R^{4}}f_{n-1}(u)\phi ((d(n),s(n),y_{n},u)du}\\ & =\frac{f_{0}(\theta )\prod_{i=1}^{n}\phi ((d(i),s(i),y_{i},\theta )}{\int_{R^{4}}f_{0}(u)\prod_{i=1}^{n}\phi ((d(i),s(i),y_{i},u)du} \end{array}$$

where $\phi ((d(n),s(n),y_{n},\theta )=p(d(n),s(n),\theta {)}^{y_{n}}(1-p(d(n),s(n),\theta ){)}^{1-y_{n}}.$

This posterior distribution is then used to make the escalation and de-escalation decision during the trials as proposed below.

### 4.3. Dual-agent dose-escalation design

The above combination-DLE model is then used in the design in Section 2.4 in place of the single-agent model. As in the single-agent setting, escalation can only occur to adjacent doses. As a consequence, no dose skipping is allowed and only escalation of one agent in the combination is permitted. In the case of equal probability for two eligible combinations, randomization is used. As the efficacy part of the dose-escalation design proposed for monotherapies considered each dose individually, the efficacy part of the combination study remains the same. Once the combination of the compounds is established to be safe, it is graduated into the efficacy part following the single-agent proposal and the same decision rules for dropping for futility and safety.

## 5. Evaluation of combination treatment design

### 5.1. Scenarios

In order to evaluate the dual-agent design, we conduct a simulation study comprising scenarios with two dose levels of agent A ( $d_{1}$ & $d_{2}$ ) and three dose levels of agent B ( $s_{1}$ , $s_{2}$ & $s_{3}$ ). We consider four dose-DLE scenarios ranging from a situation where all combinations are safe to a case where all are unsafe. Four dose-efficacy scenarios ranging from no efficacious combination to a steep monotonic within agent relationship are considered to yield 16 safety–efficacy scenarios. The dual-agent scenarios are presented in Table 4 with definitions of incorrect, undesirable, acceptable and desirable dose combinations remaining as they were previously defined for single-agent doses in Section 3.2. Here we fix cohort sizes to the previous baseline setting of $c_{1}=4$ (assigned to active dose combinations) and $c_{2}=2$ (assigned to the control) and allow controls to be shared across dose combinations. The maximum number of patients per dose combination is 72 as before.

**Table 4.** Safety and efficacy scenarios for dual-agent combinations A and B . It is assumed the control arm remains with a probability of dose-limiting event (DLE) 0.10 and a hazard ratio 1.00.

	Safety			Efficacy	
	(Probability of DLE, $p_{j}$ )			(Hazard ratio)	
		$d_{1}$	$d_{2}$	$d_{1}$	$d_{2}$
Scenario 0	$s_{1}$	0.10	0.12	1.00	1.00
	$s_{2}$	0.13	0.15	1.00	1.00
	$s_{3}$	0.15	0.18	1.00	1.00
Scenario 1	$s_{1}$	0.10	0.12	1.00	1.25
	$s_{2}$	0.25	0.30	1.25	1.50
	$s_{3}$	0.50	0.55	1.50	1.75
Scenario 2	$s_{1}$	0.15	0.30	1.00	1.50
	$s_{2}$	0.25	0.35	1.25	1.75
	$s_{3}$	0.30	0.45	1.50	2.00
Scenario 3	$s_{1}$	0.40	0.45	1.00	1.50
	$s_{2}$	0.45	0.50	1.50	1.75
	$s_{3}$	0.50	0.55	1.75	1.75

### 5.2. Parameter calibration

To define the parameters of the combination model, a calibration procedure similar to the procedure described in Section 3.3.1 was applied. Safety Scenarios 0 to 3 in Table 4 that correspond to different steepness of the combination–toxicity relationship and different locations of the target combination were used. We then choose the hyperparameters for the prior distribution of the parameters of the model, $\theta =(\theta_{1},\log\;(\theta_{21}),\log\;(\theta_{22}),\eta )$ , given in equations (9) and (10).

Given the link between the prior toxicity on the control arm and the intercept parameter $\theta_{1}$ that is common for both single-agent parameter models, we set $\mu_{1}=\mathrm{logit}({\hat{p}}_{0}^{\left(0\right)}/2)$ , where ${\hat{p}}_{0}^{\left(0\right)}=0.10$ is the DLE risk on the control, as before. To reduce the computational complexity, the covariance between all model parameters was assumed to be $\sigma_{1,21}=\sigma_{1,22}=0$ , and the variance of the slope parameters in each single-agent model is the same $\sigma_{21}=\sigma_{22}.$ Finally, we set the mean of the distribution of the interaction parameter $\mu_{\eta }=0$ to reflect that either synergistic or antagonistic effects are possible. The rest of the parameters were chosen by simulations using combinations of values on the grid, $\mu_{21}=\{-0.4,0.2,0.0,0.2\},\mu_{22}=\{-0.4,0.2,0.0,0.2\},\sigma_{1}=\{0.4,0.6,0.8,1.0,1.2\},\sigma_{21}=\sigma_{22}=\{0.05,0.15,0.25,0.35,0.45\}$ and $\sigma_{\eta }=\{0.02,0.04,0.10,0.20,1\}.$

As in the single-agent setting, the standardized doses ${\tilde{d}}_{j},{\tilde{s}}_{l}$ were calibrated in terms of the difference in the probability of DLE between the neighbouring doses. Specifically, ${\hat{p}}_{j}^{\left(0\right)}(d_{j})={\hat{p}}_{0}^{\left(0\right)}+\nu_{d}\times j,$ and ${\hat{p}}_{j}^{\left(0\right)}(s_{l})={\hat{p}}_{0}^{\left(0\right)}+\nu_{s}\times j$ , l,j=1,2,3 where $\nu_{d},\nu_{s}$ are the differences in the toxicity probabilities between doses of the first and second agents, respectively. The following values of differences were tried $\nu_{d}=\{0.025,0.05,0.075,0.10,0.125\}$ , $\nu_{s}=\{0.025,0.05,0.075,0.10,0.125\}$ . Finally, we fix $c_{\mathrm{overdose}}=0.25$ for the overdose control constraint.

Using 500 simulations under each scenario and each combination of hyperparameters, the values $\mu_{21}=0.0,\mu_{22}=0.0,\sigma_{1}=0.6,\sigma_{21}=\sigma_{22}=0.25$ , $\sigma_{\eta }=0.10,\nu_{d}=\nu_{s}=0.075$ were found to maximize the geometric mean of the proportion of correct combination selections across the scenarios.

### 5.3. Results

**Figure 4.**
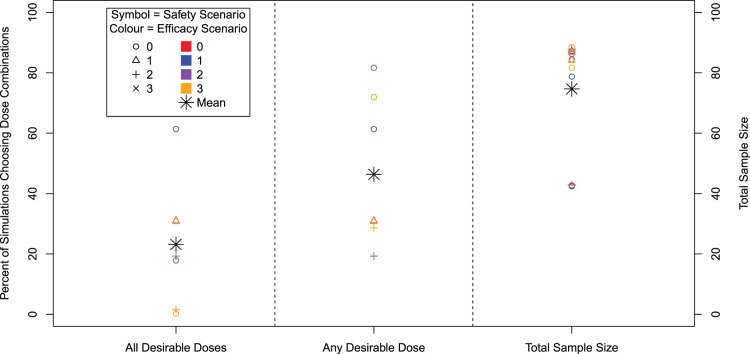
Percentage of 10,000 simulations that recommend all desirable dose combinations (left), the percentage of simulations that recommend any desirable dose combination (centre) and average total sample size. Note that only 7 out of 16 efficacy/safety scenarios contain a desirable dose combination.

For the 16 scenarios considered in the simulation study, the percentage of simulations recommending any desirable dose combination, the percentage of simulations recommending all correct dose combinations and the mean total sample size are presented in Figure 4, with further detail on individual dose combination recommendations given in Table 5. The overall type I error rate that is the percentage of simulations recommending *any* dose combination in Scenario 0–0, is 12.1%. By construction, the type I error for a given dose is controlled at 10%. In the extension from monotherapies to dual-agent therapies, some similar patterns are maintained in the results although there are some notable differences.

**Table 5.** Percentage of 10,000 simulations where each dose combination is recommended.

		Efficacy scenario							
		0		1		2		3	
Safety scenario	**0**	0.2	0.8	0.2	2.5	0.1	*5*.*1*	0.2	*5*.*0*
		0.8	2.9	2.7	*17*.*3*	2.5	**25**.**2**	*5*.*0*	**25**.**1**
		0.3	7.9	*1*.*6*	**61**.**4**	*1*.*4*	**74**.**4**	**2**.**2**	**61**.**3**
	**1**	1.1	3.0	1.2	9.5	1.2	*17*.*3*	1.3	*16*.*6*
		5.5	5.3	15.2	*23*.*3*	15.1	**31**.**0**	*28*.*1*	**30**.**7**
		0.5	0.5	1.0	1.7	1.2	1.9	1.5	1.7
	**2**	2.8	3.2	3.2	9.3	3.1	*15*.*8*	3.1	*15*.*8*
		5.7	4.0	14.7	*14*.*8*	15.0	**19**.**3**	*26*.*2*	**19**.**0**
		2.1	0.7	*8*.*6*	3.6	*8*.*2*	4.1	**11**.**1**	3.6
	**3**	6.7	1.1	6.5	2.2	6.6	2.8	6.6	2.9
		1.0	0.1	2.1	0.2	2.1	0.2	2.9	0.2
		0.0	0.0	0.1	0.0	0.1	0.0	0.1	0.0

Desirable dose combinations are highlighted in **bold** and acceptable dose combinations are highlighted in *italics*. Note that these may sum to more than 100% for each scenario as more than one dose combination can be recommended simultaneously.

It can be seen in Figure 4 that the spread of powers across scenarios for dose combinations is larger than for monotherapies, for both the selection of all and any desirable dose combinations. The maximum power of 81.7% to select any desirable dose combination is achieved in Scenarios 0 to 2, where all doses are safe and there is a steep monotonic relationship within agents for efficacy. Even though there is an extra desirable dose combination in Scenarios 0 to 3, we observe a slightly reduced power since the most efficacious dose combination has a lower hazard ratio. The power to recommend all desirable doses ranges up to 61% in Scenario 0 to 1 where only one dose is desirable, with the lowest in Scenarios 0 to 3 and 2 and 3 where the desirable dose combinations are across separate agents’ dose-escalation (i.e. to select all desirable doses requires a de-escalation in one agent and then escalation in the other agent).

Across scenarios, the mean total sample size is 75, ranging from 42 to 89, a narrower range than for the single agent. However similar to the single agent, the smaller sample sizes correspond to scenarios where all dose combinations are unsafe and therefore the trial stopped early for safety. When this is not the case, there is little variation across scenarios in terms of mean total sample size.

Table 5 shows further details of dose recommendations in the simulations. Especially of note is the emphasis on recommendations of acceptable doses. For example, in Scenarios 2 and 3, where the power to detect desirable dose combinations is low, a large proportion of simulations also recommend an acceptable dose combination. It is also clear that inefficacious and/or unsafe doses are rarely recommended.

## 6. Discussion

We introduce and evaluate the bespoke statistical design of the AGILE platform which seeks to quickly establish safe doses and potential for efficacy. The novel design utilizes a platform structure that allows the sharing of control data, includes a randomized dose-finding component and yields well-powered decisions about the activity of the treatments while controlling the type I error. We find that the design can identify potential treatments with good accuracy and show that the approach is easily extended to combinations of treatments.

The design uses a recently proposed randomized dose-finding design to ensure that differences between symptoms of COVID can be distinguished from side effects of the investigated treatment while a very simple Bayesian model is used to capture the potential efficacy of the treatments. The latter is in line with the objective of the trial: make reliable decisions about potential quickly, rather than using more complex methods that allow more precise estimation. At the same time, this approach guarantees that the whole platform structure can be simulated quickly to enable the study design to be fixed quickly.

The design has been constructed in a flexible manner using a time-to-event outcome and we based our simulations on time-to-improvement – an endpoint that has been shown recently to be a highly powered and relatively easy to collect.^12^ The platform has, however, been constructed to also be able to investigate mild disease in which case a primary endpoint used would be time-to-negative viral titres in nose and/or throat swab. Provided that the event rate in this setting is the same, we expect that the performance reported here will be similar.

In line with Yeung et al.,^28^ we have opted for separate models for safety and efficacy to allow the timing of information assessment on safety (7 days) and efficacy (28 days) to be different in order to increase the speed of the dose-escalation. At the same time, Cunanan and Koopmeiners^29^ found that in their evaluations using a joint model did not yield improved performance.

In setting up the AGILE platform and more generally when considering Phase I/II trials, several important choices, such as error rates, power and sample size need to be made. Given the exploratory nature of such studies, we believe that, in light of the small sample sizes, it is preferable to allow a somewhat larger type I error in order to achieve adequate power which will prevent missing potentially useful treatments at this early stage of development, something previously highlighted by Lindborg et al.^30^ Future development will seek to extend the design to more general prior distributions for the efficacy model and consider extensions that allow the duration of treatment to be explored in addition to dose.

## Supplemental Material

sj-pdf-1-smm-10.1177_09622802241288348 - Supplemental material for A seamless Phase I/II platform design with a time-to-event efficacy endpoint for potential COVID-19 therapies Supplemental material, sj-pdf-1-smm-10.1177_09622802241288348 for A seamless Phase I/II platform design with a time-to-event efficacy endpoint for potential COVID-19 therapies by Thomas Jaki, Helen Barnett, Andrew Titman and Pavel Mozgunov in Statistical Methods in Medical Research
