# Supplementary material for: A seamless Phase I/II platform design with a time-to-event efficacy endpoint for potential COVID-19 therapies
Source: Stat Methods Med Res. 2024 Oct 14;33(11-12):2115–30. doi: 10.1177/09622802241288348 (PMC11577684; doi:10.1177/09622802241288348)
Supplement: sj-pdf-1-smm-10.1177_09622802241288348 - Supplemental material for A seamless Phase I/II platform design with a time-to-event efficacy endpoint for potential COVID-19 therapies [file sj-pdf-1-smm-10.1177_09622802241288348.pdf]

Supporting Information for *A Seamless Phase I/II Platform Design with a Time-To-Event Efficacy Endpoint for Potential COVID-19 Therapies* by Thomas Jaki, Helen Barnett, Andrew Titman and Pavel Mozgunov

.

1 Sensitivity Analysis

In order to test the robustness of the proposed design, we conduct a sensitivity analysis on the assumptions of proportional hazards and comparison to using a binary endpoint. Since in the efficacy part, a Proportional Hazards model is assumed, this sensitivity analysis is to assess the performance of the design when the underlying efficacy data violates this assumption. Considering one of the main features of this novel design is the use of a Time-to-Event endpoint in the efficacy part, we also investigate the performance when a binary endpoint is used instead of the Time-to-Event endpoint, to illustrate the additional benefits the Time-to-Event endpoint brings.

We therefore consider the following four settings:

1. Proportional Hazards Data Generation & Time to Event Endpoint for Efficacy (Original Setting)

2. Proportional Hazards Data Generation & Binary Endpoint for Efficacy

3. Non-Proportional Hazards Data Generation & Time to Event Endpoint for Efficacy

4. Non-Proportional Hazards Data Generation & Binary Endpoint for Efficacy

These settings are all investigated for the baseline setting of $c_1 = 4$, $c_2 = 2$ and $n_c = 30$.

1.1 Non-Proportional Hazards

In the implementations in the main paper, the scenarios considered in the simulation study were under the assumption that the hazard ratio is constant over time. In these supplementary implementations for the sensitivity analysis, we instead work under the assumption that the hazard ratio is increasing over time. We consider the average hazard ratio to be the same as those in each scenario, using the restricted concordance odds definition of the average hazard ratio (defined in Martinussen and Pipper (2013)).

This is implemented by simulating recovery times from a Weibull distribution. The ratio of shape parameters between control and active arms is set to 1.5, and the rate parameter is chosen to ensure the average hazard ratio is equal to the specified value for that active dose in the scenario. In this case, the active treatment begins slightly worse than the control arm (which is constant over time), and then becomes better with time.

Note that the upper and lower boundaries for decision making in the efficacy part remain the same as those used in the main simulation study, to emulate that we are violating the assumptions of the design.

1.2 Binary Endpoint

Instead of using the recovery time as the endpoint, in this implementation we simply use a binary endpoint of recovery/no recovery.

The lower and upper boundaries for decision making in the efficacy part are calculated to be (0.167,0.838), giving a power of 66.4% with a 10% type I error rate.

1.3 Results

10,000 simulations are conducted across the 25 scenarios. Figure 1 illustrates the percentage of simulations recommending all desirable doses and any desirable doses across the scenarios for the four settings, whilst Figure 2 shows the total sample size in each case.

In scenario 1-1, the overall type I error rate in the four settings is: (11.0, 12.6, 26.0, 36.5) for settings 1-4 above respectively. Note that the definition of the null scenario is not identical to that in the main simulation study. Under the non-proportional hazards data generation, the control arm has a constant hazard ratio of 1, and the active treatment arms have an average hazard ratio of 1, which increases over time. However, this doesn't ensure that the proportion of patients recovering by 28 days is the same on active treatment as on control, and so isn't null with respect to the binary endpoint at 28 days. This goes some way to explain why the type I error rate is much higher for setting 4.

It is noticeable that when the data are generated under the proportional hazards assumption, using a binary efficacy endpoint decreases the power on average and very slightly increases the type I error rate compared to the time-to-event endpoint. The probability of recommending all and any desirable doses is greater for time-to-event efficacy endpoint under all considered scenarios, whilst the average sample size is higher under all considered scenarios.

When data are simulated under the assumption of an increasing hazard ratio, there is not much difference in terms of power on average between the time-to-event and binary endpoints, with binary very slightly higher. The probability of recommending all any desirable is similar or up to 3% in favour of the binary endpoint but for uniformly greater sample size for the binary endpoint (up to 10 patients difference) and more severely inflated type I error.

In both the binary and time-to-event settings, it can be seen that the power and type I error rate increase when generating data under non-proportional hazards compared to proportional hazards. This is most likely due to the definition of the scenarios considered. To achieve an average hazard ratio of 1.75 with an increasing hazard ratio over the 28 day follow up period, there must be a large effect at later time points. We would likely get the opposite situation (i.e. worse performance for non-proportional hazards) if we considered a decreasing hazard ratio. This is since the Cox model estimate of the hazard ratio averages the hazard ratio at the observed event times, whereas the average hazard ratio we use averages over 0-28 days (roughly) uniformly. In the increasing hazard ratio case, the event times are biased

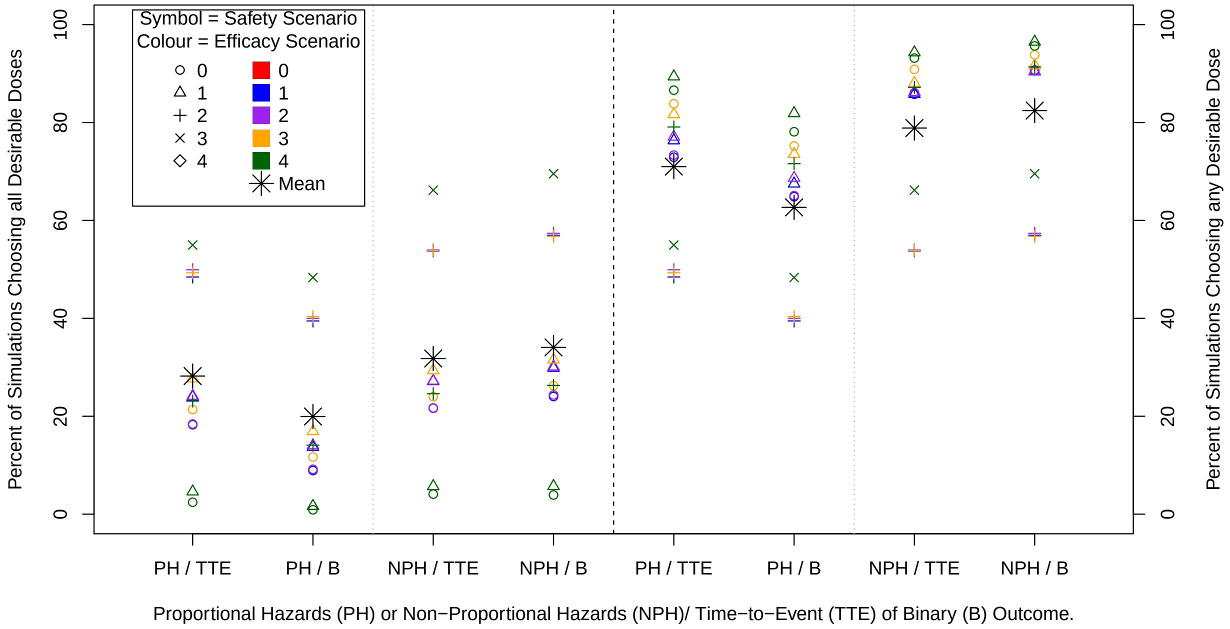

Figure 1: Percentage of simulations that recommend all desirable doses (left) and the percentage of simulations that recommend any desirable dose (right) for the sensitivity analysis. Note that only 13 out of 25 efficacy/safety scenarios contain a desirable dose.

towards later times and so the Cox hazard ratio estimate is higher than the average hazard ratio (i.e. > 1 under the "null" and > 1.75 under the alternative) and hence the inflated Type I error and power.

Overall, these additional sensitivity analyses provide evidence that the time to event approach of our novel design is superior to the use of a binary endpoint. In addition, if the proportional hazards assumption is broken by an increasing hazard ratio, there is an increase in power at the cost of an inflated type I error rate. However, using a binary efficacy endpoint inflates the type I error rate even more disproportionately to the power increase.

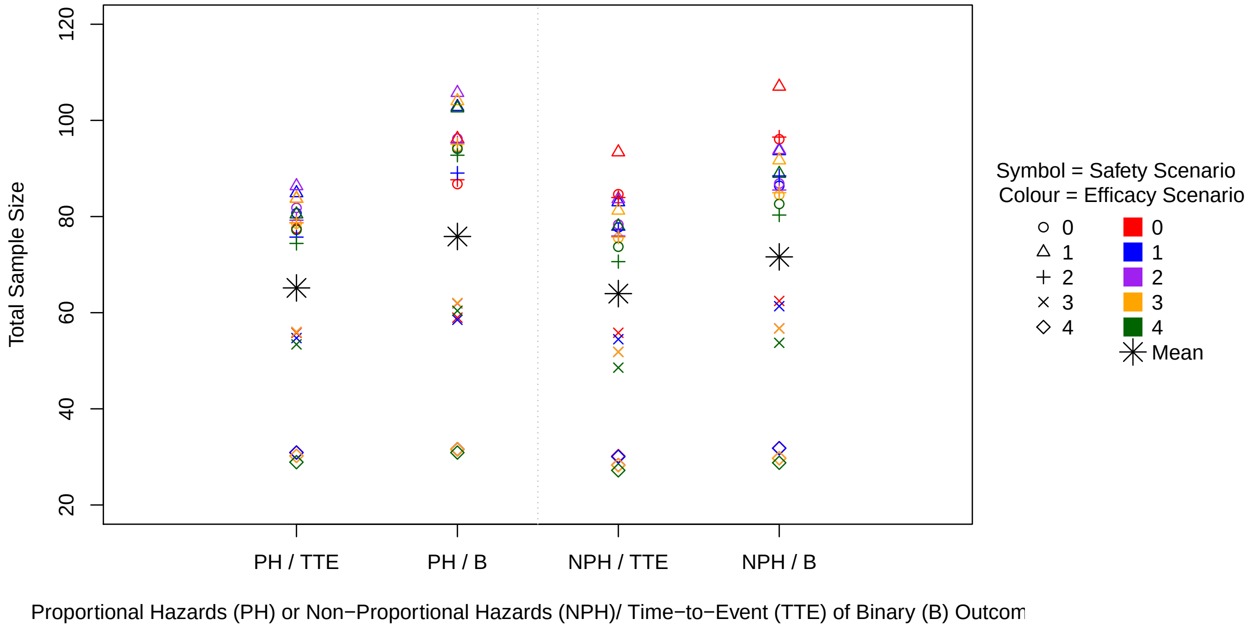

Figure 2: Average total sample size across simulations for all scenarios for the sensitivity analysis.

References

Martinussen, T. and Pipper, C. (2013). Estimation of odds of concordance based on the aalen additive model. *Lifetime Data Analysis*, 19(1):100–116.
